# Stability and changes in the distribution of *Pipiza* hoverflies (Diptera, Syrphidae) in Europe under projected future climate conditions

**DOI:** 10.1371/journal.pone.0221934

**Published:** 2019-09-04

**Authors:** Dubravka Milić, Snežana Radenković, Dimitrije Radišić, Andrijana Andrić, Tijana Nikolić, Ante Vujić

**Affiliations:** 1 University of Novi Sad, Faculty of Sciences, Department of Biology and Ecology, Novi Sad, Serbia; 2 University of Novi Sad, BioSense Institute, Novi Sad, Serbia; Instituto Federal de Educacao Ciencia e Tecnologia Goiano - Campus Urutai, BRAZIL

## Abstract

Climate change is now considered a significant threat to terrestrial biodiversity. Species distribution models (SDMs) are among the modern tools currently used to assess the potential impacts of climate change on species. *Pipiza* Fallén, 1810 is a well known aphidophagous hoverfly genus (Diptera, Syrphidae) at the European level, for which sampling has been conducted across the region, and long-term databases and geo-referenced datasets have been established. Therefore, in this work, we investigated the potential current distributions of the European species of this genus and their response to future climate change scenarios, as well as evaluated stability in their ranges and potential changes in species-richness patterns. We applied three climate models (BCC_CSM1.1, CCSM4, HadGEM2-ES) to four representative concentration pathways (RCP 2.6, RCP 4.5, RCP 6.0, RCP 8.5) for two time frames (2050 and 2070). Our results show that the distribution of most *Pipiza* species may slightly differ under different climate models. Most *Pipiza* species were predicted not to be greatly affected by climate change, maintaining their current extent. Percentages of stable areas will remain high (above 50%) for the majority of studied species. According to the predicted turnover of species, northern Europe, could become the richest in terms of species diversity, thus replacing Central Europe as the current hot spot.

## Introduction

Throughout Earth’s history, both gradual and dramatic climate changes have occurred, and many species have adapted to these alterations. Current climate change is more rapid than the rate recorded in recent history and is consequently threatening biodiversity [1]. After land-use change, current climate change is considered the second most significant threat to terrestrial biodiversity [2].

Among altered climate parameters, increased temperature is the most relevant for the distribution of living organisms [3]. However, the impact of changes in the levels of atmospheric CO_2_, UVB, ozone and nitrogen deposition, variations in moisture availability and precipitation patterns, and extreme weather events, as well as the interactions among these factors, could also be very important [4, 5, 6, 7]. Climate change, can also affect food and habitat resources, thus indirectly influencing species’ responses via shifts in distribution ranges and ecological shifts in time (changes in phenology). These phenomena would, in turn, likely lead to modifications in trophic networks and ecosystem functioning [4, 5, 8–10]. Some species can adapt through ecological processes, as evidenced by shifts towards higher latitudes or altitudes [11] and concomitant expansion/reduction of their original ranges [1], but those unable to adapt sufficiently will likely face local or global extinction [12, 13]. Empirical evidence indicates that species populations are declining and their distributions are altering much faster than in the past due to drastic contemporary changes in climatic conditions [3, 14]. In the future, the extinction risk is predicted to increase further for a large number of taxa [10, 15–17]. Climate change can also decrease genetic diversity within populations due to directional selection and rapid migration, which could in turn affect ecosystem functioning and resilience [18, 19]. Distribution changes are also associated with a wide range of anthropogenic processes, such as land-use changes, nitrogen fertilisation, pollution, over-exploitation of natural resources and introduction of invasive species [2, 5, 8, 20], affecting the response to climate change [4, 17, 21]. Certain agricultural and forestry management practices lead to habitat fragmentation, influencing the accessibility of potential habitats and the ability of species to shift their ranges [22, 23], and consequently lead to changes in distributions of many taxa at regional scales [20], reducing local terrestrial biodiversity [24].

The effects of climate change on insects are poorly understood, mainly due to limited accessibility of good quality long-term databases at a larger spatial scale for many areas or taxa [4, 21]. Research on the well-studied groups is also subject to certain limitations, such as insufficient availability of geo-referenced data at pan-European level [7], or deficient sampling procedures in certain regions. Inadequate species distributional data at all possible scales, known as the Wallacean shortfall [25], was recognised as one of the most significant impediments to the invertebrate conservation, and species distribution modelling (SDM) was proposed as a possible solution [26]. SDMs are among the most important tools currently available for assessing the potential impacts of climate change on species [27–32]. They are commonly used to forecast potential future changes in the geographic ranges of species [33–35], to estimate extinction rates [1], and to prioritise biodiversity conservation efforts [36–45]. Hoverflies (Diptera: Syrphidae) are a well-known group of insects, but until recently documentation of their current conservation status has been limited [46]. It is interesting that deceleration in the negative trend of species richness due to land use intensification has been recorded in some parts of NW Europe, whereby even in Belgium hoverflies became more diverse since 1990 [47]. Additionally, recent results show that some endemic hoverfly species in SE Europe may exhibit relatively high resilience to disturbances induced by climate change when only environmental variables are considered [48]. Nevertheless, predictions indicate that distributions of large phytophagous genus *Cheilosia* Meigen, 1822, as well as, mountainous species of genus *Merodon* Meigen, 1803 will mostly decline on the Balkan Peninsula as a result of climate change [34, 37]. However, to our knowledge, no comprehensive climate change impact modelling survey has been carried out for predaceous hoverfly species.

*Pipiza* Fallén, 1810 is one of the well known aphidophagous genus at the European level, for which sampling has been conducted across the entire region, and long-term databases and geo-referenced datasets have been created. The most recent comprehensive revision of *Pipiza* taxonomy based on both morphological and molecular evidence resolved 12 European species [49]. However, the ecology and factors shaping the distributions of these hoverflies remains largely understudied [50]. It is known that preferred habitats of *Pipiza* species mostly include forest, forest edges, tall herbs and shrubs along tracks and in open areas, whereas the predaceous larvae feed on gall-forming aphids on foliage ([51] and references therein).

Therefore, the goal of the present investigation was to: (i) investigate the potential current and future distributions of European *Pipiza* species under different climatic models and scenarios; (ii) evaluate stability in their ranges and (iii) describe and compare species-richness patterns due to climate changes.

## Material and methods

### Species occurrence data

The material used in the present study was determined by Ante Vujić. It is deposited in the museums, universities and private collections listed in S1 Appendix.

**Fig 1:**
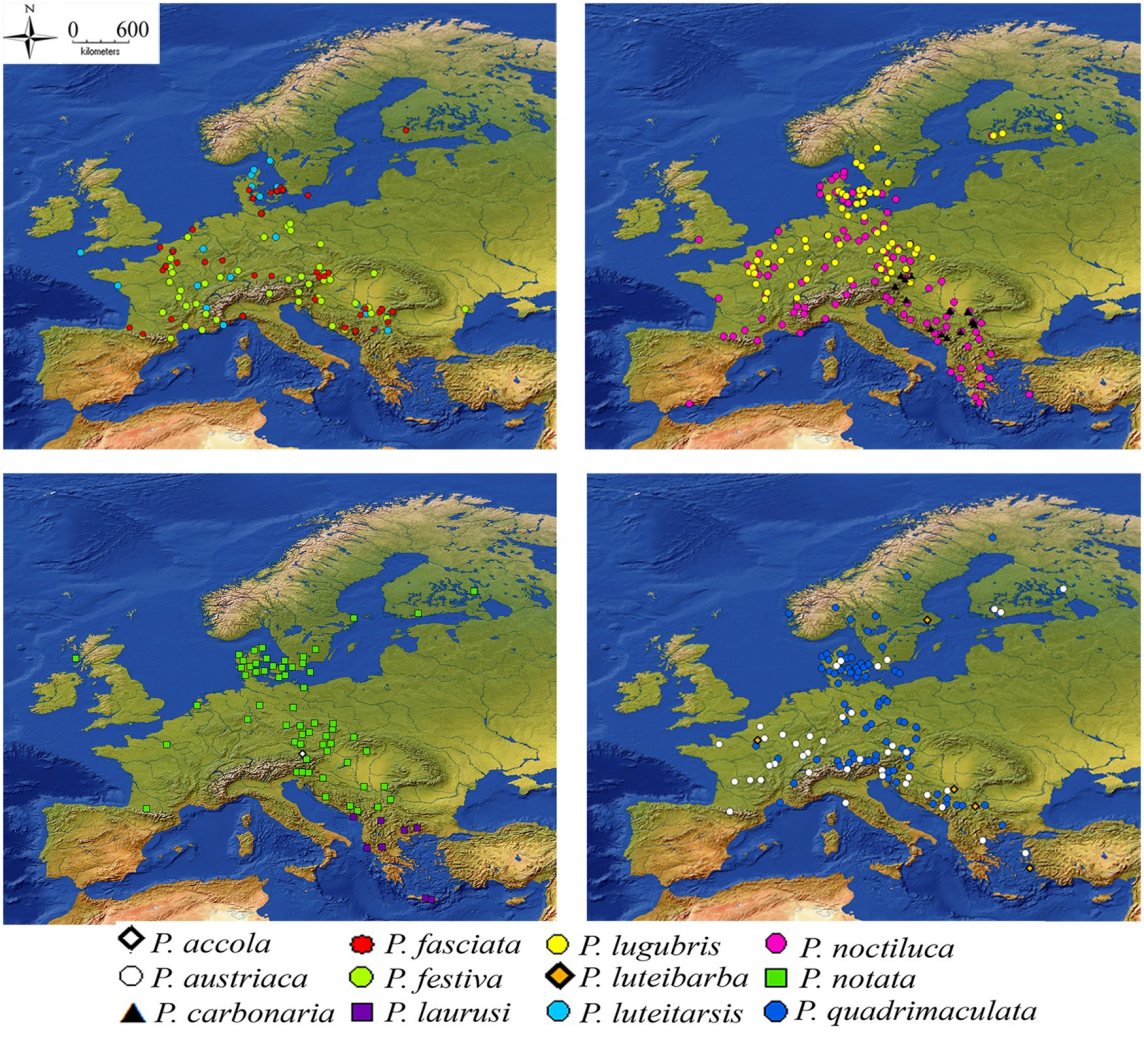
Occurrences of each *Pipiza* species in Europe.

We extracted geo-referenced occurrence data for 12 European *Pipiza* species for the 1960–2013 period: *P*. *accola* Violovitsh, 1985, *P*. *austriaca* Meigen, 1822, *P*. *carbonaria* Meigen, 1822, *P*. *fasciata* Meigen, 1822, *P*. *festiva* Meigen, 1822, *P*. *laurusi* Vujić & Ståhls, 2013, *P*. *lugubris* (Fabricius, 1775), *P*. *luteibarba* Vujić, Radenković & Polić, 2008, *P*. *luteitarsis* Zetterstedt, 1843, *P*. *noctiluca* (Linnaeus, 1758), *P*. *notata* Meigen, 1822, *P*. *quadrimaculata* (Panzer, 1804) (Fig 1).

Duplicate records were removed prior to the analysis. For reducing sampling bias, we applied a species occurrence record thinning procedure using the function ‘thin’ in the package *red* [52] in R [53], where we used a threshold of 0.01 of the maximum distance between any two points. The procedure is explained in detail in Miličić et al. [35]. This procedure sequentially removes occurrence points that are closer to each other than a predefined distance in order to minimise environmental and geographical bias. The ‘thin’ function returns a dataset with the maximum number of records for a specified thinning distance when run for a sufficient number of iterations. After data processing, species with fewer than ten spatially distinct records (*P*. *accola*, *P*. *laurusi* and *P*. *luteibarba*) were removed. Occurrences of species used in this study are available at https://cbbc.pmf.uns.ac.rs/wp-content/uploads/2019/08/Pipiza_occurence-points.xlsx

### Environmental variables

Current climate data were obtained from the WorldClim database, which contains interpolations of global temperature and precipitation at a 2.5 arc minute resolution [54]. Future climate scenarios were based on four Representative Concentration Pathways (RCPs, [55]), namely RCP 2.6, RCP 4.5, RCP 6.0 and RCP 8.5.

RCPs are developed by the Intergovernmental Panel on Climate Change [56]. The four aforementioned pathways were chosen because RCP 2.6 denotes the lowest GHG concentration pathway, based on the premise that radioactive forcing (global energy imbalance) levels will reach 3.1 W/m^2^ by mid-century and will decline to 2.6 W/m^2^ by 2100, while global mean temperatures will increase by 0.2–1.8°C [57]. On the other hand, in RCP 4.5, denoted as a stabilisation scenario, by 2100, the total radioactive forcing would reach 4.5 W/m^2^ and would stabilise thereafter due to the adoption of technologies and strategies aimed at reducing GHG emissions. In this scenario, global mean temperatures will increase by 1.0–2.6°C [58]. In RCP 6.0, stabilisation by 2100 is also projected, reaching 6.0 W/m^2^ [59]. Finally, RCP8.5 was considered in the present study as it is a pessimistic scenario, according to which 1,350 ppm CO_2_ and 2.6–4.8°C temperature increase would occur by 2100 [55]. Relative bioclimatic variables were downloaded from http://www.worldclim.org for 2050 (averaged for 2041–2060) and 2070 (averaged for 2061–2080) time frames, according to the Beijing Climate Center climate system model—BCC_CSM1.1 [60], The Community Climate System Model—CCSM4 [61] and Hadley Global Environment Model 2 Earth System configuration—HadGEM2-ES [62] at a resolution of 2.5 arc minutes.

Topographic variable was represented by altitude derived from a 30 m digital elevation raster [63]. Elevation data for each location were obtained from a 30m digital elevation raster [63] using DivaGis software [64]. Using Statistica^®^ for Windows [65], a box plot was generated to graphically display the positions of investigated specimens of each species in altitudinal range.

Habitat variables were obtained from Corine Land Cover (CLC) [66]. The standard CLC nomenclature includes 44 land cover classes. Land cover variables were transformed into different land cover categories within every grid cell in Arc-View GIS 10.1. We chose three habitat types and calculated their areas in each 5x5 km cell. Our habitat classification generally matched the second or the third level of the Corine classification. We combined the Natural Grassland and Pastures categories into the *grassland variable*, whereas Broad-leafed forests, Coniferous forests and Mixed forests were merged to *forest variable*, and *agricultural land variable* was created by combining Permanently and Non-irrigated arable land, Vineyards, Fruit trees and berry plantations. Moreover, we calculated Euclidean distance from forest surface covering more than 40km^2^ area. All variables have a spatial resolution of 2.5 arc minutes.

Bioclimatic variables, elevation, grid cell proportion covered by grassland, forest and agricultural land, as well as distance from forest were first subjected to multicollinearity test, whereby variance inflation factors (VIF) analysis was performed for all species in the R platform [53] using the *usdm* package [67]. As the obtained VIF values indicated high level of collinearity we sequentially eliminated the covariate with the highest VIF from the set, before recalculating the VIFs, repeating this process until all VIFs values declined below 3. The remaining variables were incorporated into the models of the current potential distribution of each species (S1 Table).

### Modelling procedure

Current and future species distributions were modelled using a maximum entropy algorithm implemented in the MaxEnt software [68, 69]. Maxent is a machine-learning technique that can be adopted to calculate the potential geographic distribution of species based on the probability distribution of maximum entropy. It has been widely used in ecological studies with over 1.000 applications published since 2006 [70, 71]. The software can be applied to records that have not been collected as a part of systematic biological surveys, which is highly advantageous when processing data based on museum collections [72–74]. In comparison to other similar methods that yield predictions of species distribution (Classification And Regression Trees CART [75], Bioclim [76], Generalized Linear Models − GLM [77], and Artificial Neural Networks [78]), MaxEnt is an efficient method for modelling species distributions using presence-only data. It can also be applied for processing complex interactions between response and predictor variables [27]. Available evidence further indicates that it can be applied to both categorical and continuous data variables [72], and it efficiently transfers the model projections to another geographical area [79].

MaxEnt models were generated as a part of the current investigation to forecast present and future geographical distribution of the selected species. To validate model performance with respect to the current potential distribution of each selected species, k-fold cross-validation was conducted for partitioning data into training and testing sets, whereby 75% of the occurrence data for each replicate was used to calibrate the model, and the remaining 25% was used to evaluate the model using the area under the curve (AUC) of the receiver operating characteristic (ROC). In general, AUC values <0.7 are indicative of an inaccurate model (with no better than random performance), whereas values >0.9 indicate a high model accuracy [68, 72]. To control model complexity and define the best combination of MaxEnt’s feature classes and regularisation multipliers, we initially used the ENMeval package in R [80]. We tested 48 model candidates by using all combinations of feature types (L, LQ, LQH, LQHP, LQHPT) and regularisation multipliers (from 0.5 to 4), along with the lowest Akaike’s information criteria (AICc) values (ΔAICc = 0), to select the best model candidate [80–82].

The results yielded by the best model candidate were subsequently utilised to develop 25 different model projections for nine species, with one current and eight future projections of potential distribution for each species, resulting in 225 models in total. For the evaluation of the best model candidate prediction, in addition to the aforementioned test statistics (ROC and delta AIC), we considered low omission rates and Kappa statistics. We applied the "maximum specificity plus sensitivity" threshold, which balances both omission and commission errors, to transform each separate continuous suitability raster into binary maps representing areas of potential suitability for species occurrence [83, 84].

Changes in species ranges were calculated by comparing the percentages of areas that were gained or lost under different climate models and RCP climate change scenarios. Furthermore, each species’ map of current potential distribution and their respective future potential distributions (under all four RCP scenarios), were superimposed to assess: (1) the stable area (the area inhabited by a species today and under all future scenarios); (2) the potential new area (area not currently inhabited, but likely to be inhabited under all future scenarios); and (3) the lost area (area inhabited at the present time, but uninhabited under all future scenarios).

### Species richness—Percentage turnover

Distribution maps of overall species richness for the present and future (2050 and 2070) under all four RCP climate scenarios were produced by summing all of the predicted distribution maps for individual species.

Species turnover (T) is defined as changes in the number of species in a specific location and is considered an appropriate measure of altered species composition in response to climate change at regional to continental levels [85–87]. It is calculated as the difference between current and future species composition [86]. Thus, a turnover value of 0 indicates that the predicted future assemblage will remain the same as the current one, whereas a turnover value of 100 indicates that the assemblage will change completely. To determine T, we calculated the number of species predicted to lose suitable habitat (i.e. the number of species lost—L) and the number of species predicted to gain suitable habitat (i.e. the number of additional species—G) for each grid cell. The percentages of species turnover per grid cell between the present day and either 2050 or 2070 was determined by applying the following expression, T = 100 (L + G)/(SR + G), where SR is the current predicted species richness. All analyses were carried out in ArcGIS vs. 10.1.

### Ethics statement

None of the collected hoverfly species are red listed, endangered, threatened or considered to be endangered in Europe. Similarly, no species that were collected as a part ofthe present study are ranked in any IUCN list or protected by CITES. All specimens were collected on state-owned property. The collection of these species is not subjected to restriction by law and does not require collecting permits in these countries.

## Results

ENMeval package results suggested the usage of hinge feature for most *Pipiza* species (S2 Table). Model performance for individual species was good, as indicated by AUC and AUCTest values ˃ 0.7 for all species.

**Fig 2:**
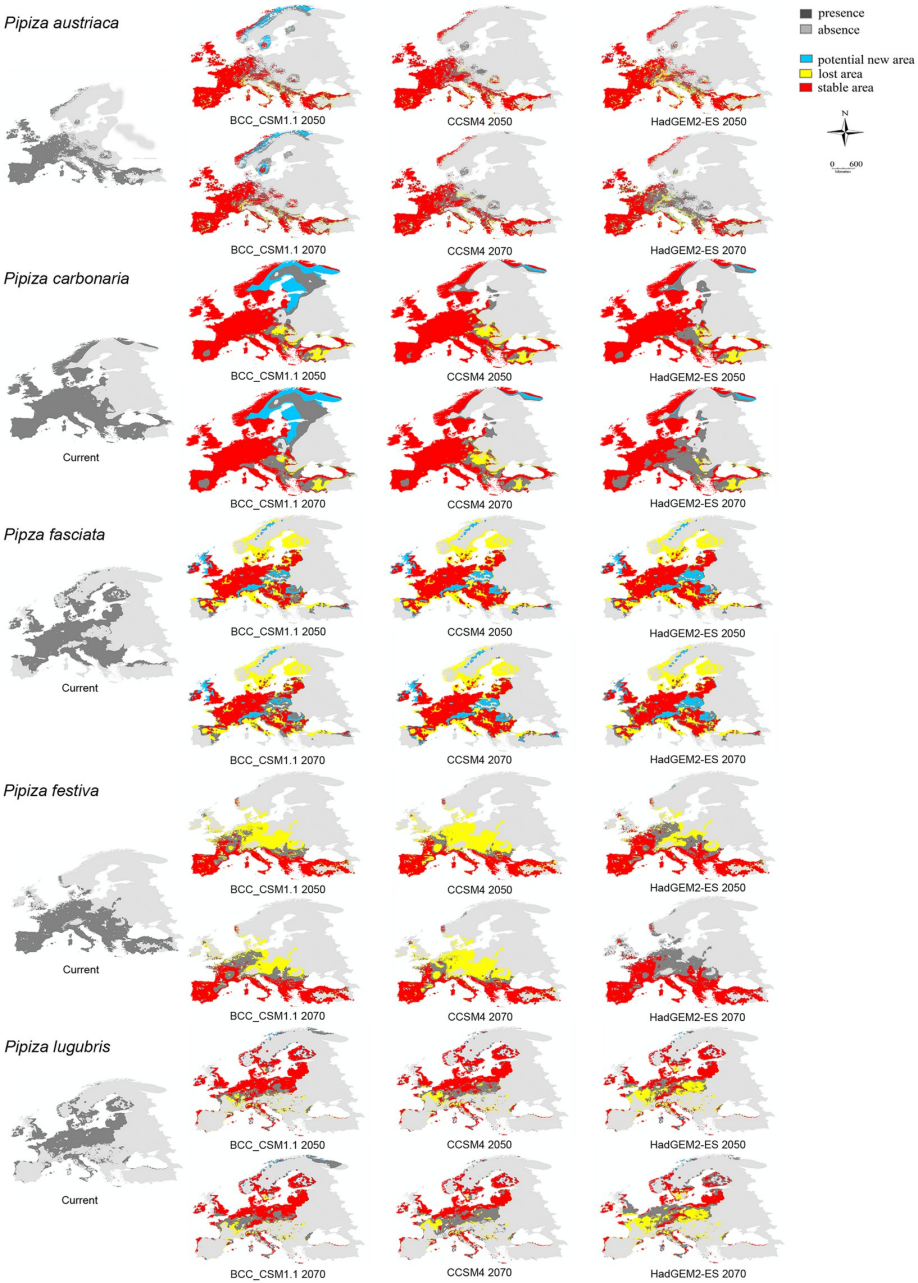
Modelled species distributions under current climate and future climatic models and all scenarios for: *P*. *austriaca*, *P*. *carbonaria*, *P*. *fasciata*, *P*. *festiva*, *P*. *lugubris*.

**Fig 3:**
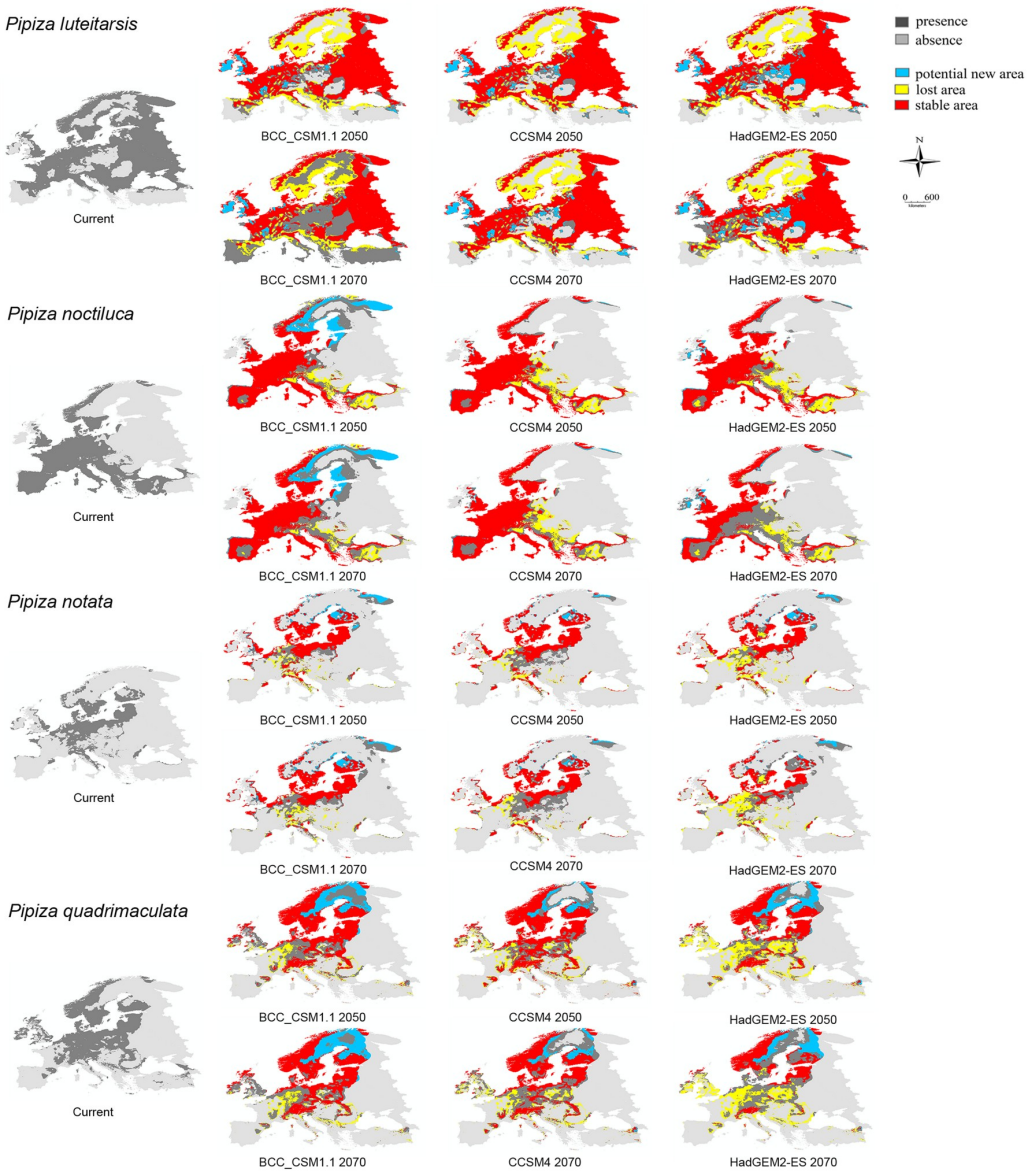
Modelled species distributions under current climate and future climatic models and all scenarios for: *P*. *luteitarsis*, *P*. *noctiluca*, *P*. *notata*, *P*. *quadrimaculata*.

Most of the *Pipiza* species assessed as a part of the current investigation are broadly distributed throughout Europe, from Fennoscandia through central and southern Europe (excluding the Iberian Peninsula that only harbours one *Pipiza* species, i.e. *P*. *noctiluca*). *P*. *carbonaria* is found only in the Balkans and Austria, and *P*. *laurusi* only occurs in SE Europe (Montenegro, FYR Macedonia and Greece) (Figs 1–3).

According to the available occurrence data, the altitudinal ranges for *Pipiza* species were very wide, ranging from 10 to approximately 2.700 metres above sea level (m.a.s.l.) (S1 Fig). However, elevation hardly explained the current distribution of *Pipiza* species in Europe (Table 1).

**Table 1:** Percentage contributions of the studied variables to the modelled distribution of *Pipiza* species.

Environmental variables	Pa	Pc	Pfa	Pfe	Plg	Plu	Pnc	Pn	Pq
Elevation	0.1	-	-	-	-	-	0.1	-	23.1
Annual Mean Temperature (BIO1)	-	-	0.1	0.1	-	-	-	-	-
Mean Diurnal Range (BIO2)	0.1	0.1	-	3.9	47.2	0.1	1.7	54.2	23.6
Temperature Seasonality (BIO4)	5.1	75.1	-	-	-	-	90.4	-	-
Mean Temperature of Wettest Quarter (BIO8)	0.1	-	0.1	-	12.9	0.1	0.1	9.4	10.7
Mean Temperature of Driest Quarter (BIO9)	43.6	-	-	74.4	-	-	-	-	36.7
Precipitation of wettest month (BIO13)	-	-	-	1.1	-	-	-	-	0.1
Precipitation Seasonality (BIO15)	50.7	24.6	50.4	0.1	4.0	73.6	0.1	4.9	0.1
Precipitation of Warmest Quarter (BIO18)	-	-	0.1	-	3.0	-	0.1	1.2	-
Distance from forest	0.1	0.1	-	20.3	-	0.1	0.1	0.2	-
Proportion of the grid cell covered with grassland	0.1	-	10.1	-	5.3	16.4	1.2	4.9	-
Proportion of the grid cell covered with forest	0.1	0.1	0.1	0.1	7.6	9.7	0.2	0.5	0.1
Proportion of the grid cell covered with agricultural land	-	-	39.1	-	19.9	-	6.0	24.8	5.6

Pa—*P*. *austriaca*, Pc—*P*. *carbonaria*, Pfa—*P*. *fasciata*, Pfe—*P*. *festiva*, Plg—*P*. *lugubris*, Plu—*P*. *luteitarsis*, Pnc—*P*. *noctiluca*, Pn—*P*. *notata*, Pq—*P*. *quadrimaculata*

Although all habitat variables (proportion of the grid cell covered by forest, grassland and agricultural land, and distance to forest) affected the potential distributions of all species, their explanatory power was limited. Proportion of the grid cell covered with agricultural land had the greatest contribution among all investigated habitat variables for *P*. *fasciata*, *P*. *notata*, *P*. *lugubris*, *P*. *noctiluca* and *P*. *quadrimaculata* at 39.1, 24.8, 19.9, 6.0, and 5.6%, respectively. The ranges of all investigated species were mostly influenced by climatic variables. Precipitation Seasonality (BIO15) was the most significant contributor to the distribution of *P*. *austriaca*, *P*. *fasciata* and *P*. *lugubris*, whereas *P*. *carbonaria* and *P*. *noctiluca* distributions were strongly predicted by temperature seasonality (BIO4). Mean diurnal range (BIO2) exerted the greatest influence on the distributions of *P*. *lugubris* and *P*. *notata*, while mean temperature of driest quarter (BIO9) was the most influential on *P*. *festiva* and *P*. *quadrimaculata* distribution (Table 1). The contributions of other climatic variables were below 10 for all investigated species.

The study findings indicate that the species distributions projected by the CCSM4 and HADGEM2-ES climate models and scenarios were similar (S3 Table, Figs 2 and 3), while a slight variation was obtained when BCC_CSM1.1 was applied. According to the results yielded by CCSM4 and HADGEM2-ES, all *Pipiza* species are predicted to lose a part of their range, with the smallest loss of 0.49% projected for *P*. *notata* for 2050 by the CCCM4 climate model (RCP 2.6) and the highest (59.55%) forecast by the HADGEM2-ES for 2070 for *P*. *lugubris* (RCP 8.5). Only three species *P*. *carbonaria*, *P*. *noctiluca* and *P*. *notata* were predicted to extend their range in the future according to the BCC_CSM1.1 climate model for both 2050 and 2070, which forecast range loss for almost all investigated *Pipiza* species. The only exception to these trends was noted for *P*. *quadrimaculata* predicted to extend its range in northern Europe by 2050, after which its distribution was forecast to decline by 2070 (with the losses confined mostly to central and eastern Europe). Based on the CCCM4 and HADGEM2-ES forecasts, *P*. *quadrimaculata* may reduce its range.

For most of the examined *Pipiza* species high percentages of stable area (over 50%) were predicted. The only exceptions are *P*. *festiva* according BCC_CSM1.1 and CCSM4, *P*. *quadrimaculata* based on the HADGEM2-ES prediction for both years (Table 2, Figs 2 and 3) and *P*. *lugubris* and *P*. *notata* for the 2070 year according HADGEM2-ES climate model. The greatest percentage of lost area (up to 44%), mostly from central and east Europe, was noted for *P*. *festiva* (Fig 2). The lowest area loss, mainly on the Balkan, Iberian and Apennine Peninsulas, was recorded for *P*. *austriaca*, according to BCC_CSM1.1 and CCSM4 climate models (Table 2, Fig 2). The highest proportion of a potential new area by 2050 in Fennoscandia, western part of Great Britain, the Alps and eastern Europe was predicted for *P*. *fasciata* by the CCSM4 and HADGEM2-ES climate models, while BCC_CSM1.1 predicted the highest proportion of a potential new area for *P*. *carbonaria* for the same year.

**Table 2:** Percentages of potential new, lost and stable areas for each *Pipiza* species in Europe based on the BCC_CSM1.1, CCSM4 and HadGEM2-ES climate model predictions and all scenarios. Bold numbers indicate the smallest and highest percentage.

Species & coresponding climate models	Potential new area		Lost area		Stable area	
	2050	2070	2050	2070	2050	2070
BCC_CSM1.1						
*P*. *austriaca*	7.93	6.95	**5.30**	**4.97**	73.51	73.91
*P*. *carbonaria*	**20.46**	**19.47**	7.23	4.39	80.76	78.82
*P*. *fasciata*	11.87	10.45	34.03	33.41	54.94	50.32
*P*. *festiva*	0.0034	0.0070	35.25	22.79	**44.43**	**48.25**
*P*. *lugubris*	2.46	2.26	7.90	8.38	69.61	63.80
*P*. *luteitarsis*	4.69	3.88	20.83	21.25	68.08	62.38
*P*. *noctiluca*	18.34	17.43	11.16	8.14	71.47	70.16
*P*. *notata*	12.65	11.71	9.64	9.65	71.96	68.18
*P*. *quadrimaculata*	17.88	22.71	10.14	11.03	63.38	60.81
CCSM4						
*P*. *austriaca*	0.13	0.13	**4.44**	**4.41**	77.94	69.88
*P*. *carbonaria*	1.28	1.89	8.04	7.20	81.82	76.58
*P*. *fasciata*	**14.00**	**14.81**	33.12	32.33	61.91	61.39
*P*. *festiva*	0.00044	0.00044	**44.28**	**39.72**	**43.95**	**44.26**
*P*. *lugubris*	2.00	1.53	10.53	10.12	65.24	54.86
*P*. *luteitarsis*	5.35	6.11	19.61	18.24	73.02	74.28
*P*. *noctiluca*	0.63	1.09	13.27	13.72	76.06	69.54
*P*. *notata*	4.91	4.49	9.63	9.35	69.42	61.29
*P*. *quadrimaculata*	8.37	8.83	12.85	13.81	64.18	53.64
HadGEM2-ES						
*P*. *austriaca*	0.30	0.23	10.30	7.79	71.94	52.09
*P*. *carbonaria*	1.69	2.23	5.85	4.09	81.85	65.12
*P*. *fasciata*	**15.16**	**13.26**	32.028	32.83	60.21	53.32
*P*. *festiva*	0.038	0.061	18.38	0.064	56.99	62.41
*P*. *lugubris*	2.35	1.76	25.18	27.29	51.83	**33.84**
*P*. *luteitarsis*	7.59	6.64	19.92	20.96	71.63	64.80
*P*. *noctiluca*	2.02	2.60	14.24	10.68	71.43	51.25
*P*. *notata*	8.00	4.05	18.90	23.35	61.38	**42.18**
*P*. *quadrimaculata*	15.01	13.29	27.9	29.89	**48.54**	**33.52**

**Fig 4:**
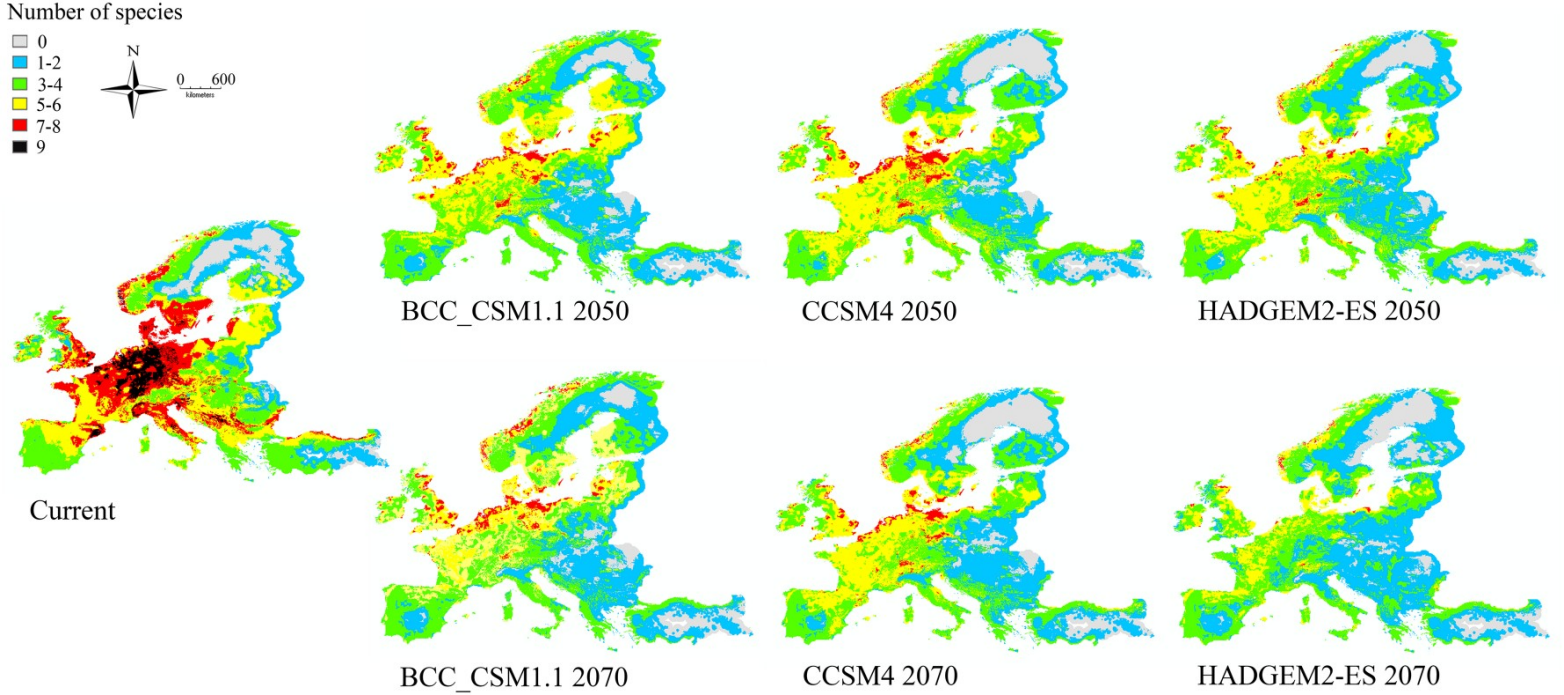
Spatial distributions of predicted current and future (2050 and 2070) species richness based on the BCC_CSM1.1, CCSM4 and HadGEM2-ES climate models.

Areas predicted to have the highest number of species (nine species) under current conditions are southern parts of Fennoscandia, small parts of Great Britain, central Europe, central part of Apennines Peninsula, small areas in the Pyrenees and on the Dinaric Mountains. Under the future climate scenarios for both 2050 and 2070, North Europe and the Alps are predicted to become the most species-rich area, even though a diminishing trend between 2050 and 2070 was predicted by all climate models (Fig 4).

**Fig 5:**
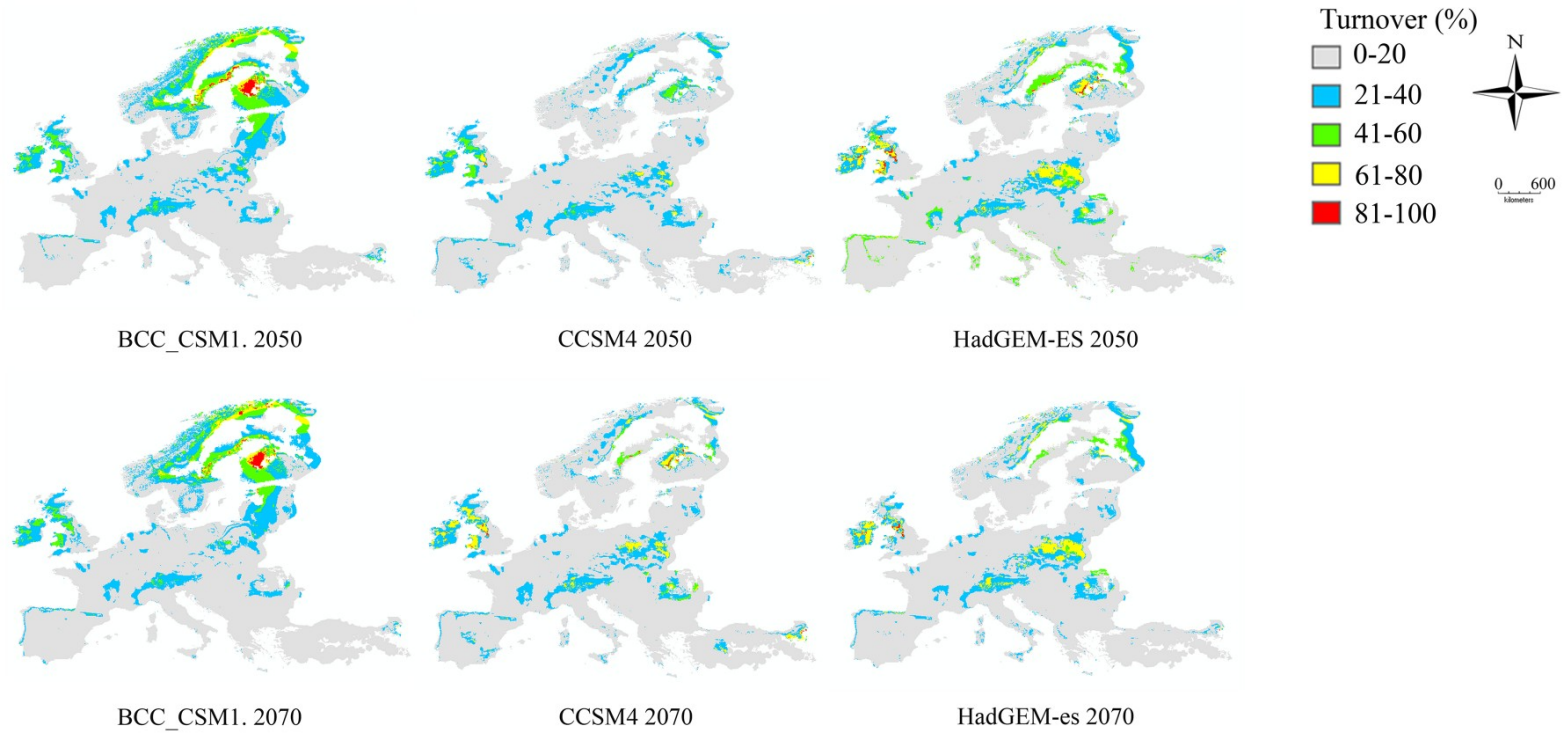
Percentage turnover of *Pipiza* species for 2050 and 2070 across all RCP scenarios based on the BCC_CSM1.1, CCSM4 and HadGEM2-ES climate models.

Percentage species turnover was similar for 2050 and 2070 (Fig 5). Across all climate models and scenarios, as well as years, Fennoscandia would be the most affected by the species turnover. Slightly lower percentage of turnover (61–80%) was also predicted in eastern Europe. These results indicate increased changes in species assemblages in the northern most part of Europe in the future. These predicted changes are less pronounced according to the CCSM4 and HADGEM2-ES models.

## Discussion

We forecasted the effect of climate change on the distribution of nine *Pipiza* species in Europe using different climate models and scenarios for two future frames (2050 and 2070). The performance of all tested models when applied to individual species was good. Wisz et al. [88] demonstrated that the lowest AUC is typically associated with species with the lowest number of records (10–30). In general, our results support these findings. However, the AUC obtained for *P*. *festiva* and *P*. *noctiluca* was unrelated to sample size. Moreover, lower number of predictor variables (five and six, respectively) was noted for species for which the sample size was smaller (*P*. *carbonaria* and *P*. *luteitarsis*), which could compromise the potential for discerning species–environment relationships.

The results obtained via BCC_CSM1.1 and the two remaining climate models differed with respect to the number of species with enlarged distribution. According to BCC_CSM1.1, three (plus *P*. *quadrimaculata* partly) of nine species may extend their range by up to 16%, whereas CCSM4 and HadGEM2-ES predict reduction in the ranges of all investigated species. This incongruence in findings may be in part related to the differences in climate sensitivity characterising these models [89–92].

Climate-related variables, especially temperature, are the most significant contributors to the current distribution of majority of the species, denoted in our investigation as: Mean Diurnal Range (BIO2), Temperature Seasonality (BIO4) and Mean Temperature of Driest Quarter (BIO9). Dixon et al. [93] demonstrated that, in insects, developmental rates are affected by temperature levels within a given thermal window. The present findings could be interpreted through the biological lens, whereby we argue that the temperatures in the first months of spring likely affect the final stage of larval development and adult emergence in *Pipiza* species. Additionally, temperature can act indirectly by affecting phenology, abundance and quality of visiting plants that are food resource for adults, as well as for aphids that are larval prey. Although Nikolić et al. [50] concluded that most of the *Pipiza* species prefer forests habitats, our results showed that climate would have the most influential impact on their distribution. Moreover, climate change could fundamentally alter the composition, structure, and biogeography of forests in many regions [92]. In Europe, forest mortality due to dry and warm conditions, coupled with biotic stressors, has already increased mortality of deciduous and coniferous species [94–96].

Despite projected potential range reduction, percentages of stable area remain high (above 50%) for most studied *Pipiza* species, with the exception of in *P*. *festiva* (as indicated by BCC_CSM1.1 and CCSM4), *P*. *quadrimaculata* based on the HADGEM2-ES prediction for both years and *P*. *lugubris / P*. *notata* for the 2070 year according HADGEM2-ES climate model. This can be explained by less expressed specific requirements, as most of the *Pipiza* species, while exhibiting preference for forest habitats, can be found across wide altitudinal range, while some also thrive in agroecosystems.

In general, potential new area for all *Pipiza* species is relatively modest (up to 23%). According to all climate models, potential new area would emerge in northern Europe, and this region would become the most species-rich on the continent. Similar patterns in range shift have been already recorded for mountainous species of the large hoverflies genus *Cheilosia* [37] and other insect groups in Europe. Parmesan et al. [97] observed that more than half of investigated European butterfly species have expanded their ranges to the north, as well as dragonflies and damselflies in Great Britain [98]. In a more recent SDM study conducted by Fourcade et al. [23] using a butterfly as model species, it was predicted that its climatically suitable habitats may extended north of its realised European range. Extensive body of empirical evidence indicates that European butterflies are highly vulnerable to climate change, as most species are expected to shift their distributions considerably northwards [99], with the northern European species likely to be the most vulnerable [100]. However, the range shift of lepidopteran species in response to future climate change may be limited by future land use and the adaptability of its host plants [23, 32, 101–103]. Available evidence further indicates that certain critical characteristics, such as low dispersal ability, degree of habitat specialization and hostile landscapes, may also play a role [99, 100, 104]. On the other hand, Hof and Svahlin’s [105] investigation of 30 prospective insect pest species (Coleoptera and Lepidoptera) predicted large increases in their future distribution in Scandinavia, which may result in outbreaks in new areas.

Our results show that the region predicted to have the greatest richness of *Pipiza* species under all considered climate models and scenarios for the 2050 and 2070 horizons overlaps with a potentially new area in North Europe, thus replacing Central Europe as the current hot spot, making it an important centre of diversity for European *Pipiza* in the future. If species of this genus would be able to colonize new suitable areas far outside their current distribution, largely depend on their dispersal ability. Until now there is no known migratory *Pipiza* species and not enough data for this kind of estimation. Based on their comprehensive study on European bumblebees, Rasmont et al. [106] forecast a shift of suitable areas due to climate change for many species, predicting expansion toward northern Europe (especially to Fennoscandia). In the northernmost localities, the authors predicted maintenance or even an increase in the number of species. Even though the most sensitive species might be at risk in these regions, a gain is likely due to colonization from the south [106].

When these findings are compared to the "previously reported results" for the two large European hoverfly genera, both of which are phytophagous, it can be concluded that each has specific response to climate change. In general, genus *Cheilosia* is better adapted to cold and is thus more affected, whereas *Merodon* exhibits preference to warm climate, as indicated by its south-eastern Mediterranean hot spot. Hence, in *Merodon* only high mountain species may potentially be negatively influenced by climate change, shifting their range more to the north, while others may even expand their distribution due to global warming [34, 37]. Genus *Pipiza* has a habitat preference similar to *Cheilosia*, as both mostly comprise of typical forest species, but with fewer exclusively high mountain species. Thus, most probably this is the reason for mild response of *Pipiza*, positioned in between these two phytophagous genera. On the other hand, the lack of available data about the larval host plants / prey and their distributions constrained our ability to investigate their role within the modelling framework adopted for these three genera. Additionally, Harrington et al. [107] showed that advance in first flight seasonal record of European aphids could be expected as the temperature increases. This event is also likely to influence earlier emergence of *Pipiza* species, but would most probably not affect the size of the stable area, which would give this aphidophagous genus a greater chance of survival despite climate changes.

The general pattern in different insect groups includes range shifts to northern latitudes and higher elevations as a result of climate change, as predicted for most well-studied groups of organisms [8]; however future insect distributions will be governed by degree of specialization (host and habitat range) [5, 7, 108]. Although climate is expected to be the dominant factor affecting the distribution of species at the European scale [109], the degree to which different insects will be affected by climate change may be influenced by habitat availability [21, 110] and food resources [4, 9, 109, 111]. Thus, to gain a better understanding of the sensitivity of the *Pipiza* species to future climate conditions, other influential variables, such as competition, food resource and larval development, that are currently unknown, should be investigated as a part of future research.

Although accurate predictions of climate and species distributions might not be entirely achievable, appropriate strategies for applying existing knowledge and bioclimatic modelling can improve our understanding of the possible effects of climate change on biodiversity, especially given continued advancements in SDM [112, 113]. Despite some notable limitations, still it is better to use results from SDMs for conservation planning than implementing conservation measures in response to climate change without any scientific basis [37].

## Supporting information

S1 Fig Variability plot of altitudinal gradients for species of the genus *Pipiza*. (TIF)

S1 Appendix List of collections were the material used in this study was deposited. (DOCX)

S1 Table Numbers of records for analysed species and respective variables used to develop the model and their percentage contribution. (DOCX)

S2 Table Model comparison based on Akaike Information Criteria (AICc). (DOCX)

S3 Table Percentages of range reduction/expansion relative to current distributions for species of the genus *Pipiza* in Europe based on the BCC_CSM1.1, CCSM4 and HadGEM2-ES climate model predictions and for different climate change scenarios. (DOCX)

## References

[1] Thomas CD, Cameron A, Green RE, Bakkenes M, Beaumont LJ, Collingham YC, et al Extinction risk from climate change. Nature. 2004; 427: 145–148. 10.1038/nature02121 14712274

[2] Sala OE, Chapin FS, Armesto JJ, Berlow E, Bloomfield J, Dirzo R, et al Global Biodiversity Scenarios for the Year 2100. Science. 2000; 287 (5459): 1770–1774. 10.1126/science.287.5459.1770 10710299

[3] Chen IC, Hill JK, Ohlemüller R, Roy DB, Thomas CD. Rapid Range Shifts of Species Associated with High Levels of Climate Warming. Science. 2011; 333 (6045): 1024–1026. 10.1126/science.1206432 21852500

[4] Menéndez R. How are insects responding to global warming? Tijdschr Entomol. 2007; 150: 355–365.

[5] Wilson RJ, Davies ZG, Thomas CD. Insects and Climate Change: Processes, Patterns and Implications for Conservation. In: Stewart AJA, Lewis OT, New TR, editors. Insect Conservation Biology. Proceedings of the Royal Entomological Society’s 22nd Symposium. CAB International Publishing; 2007. pp. 245–279.

[6] Lindner M, Maroschek M, Netherer S, Kremer A, Barbati A, Garcia-Gonzalo J, et al Climate change impacts, adaptive capacity, and vulnerability of European forest ecosystems. For Ecol Manage. 2010; 259: 698–709. 10.1016/j.foreco.2009.09.023

[7] Barredo JI, Strona G, de Rigo D, Caudullo G, Stancanelli G, San-Miguel-Ayanz J. Assessing the potential distribution of insect pests: case studies on large pine weevil (*Hylobius abietis* L) and horse-chestnut leaf miner (*Cameraria ohridella*) under present and future climate conditions in European forests. EPPO Bulletin. 2015; 45 (2): 273–281. 10.1111/epp.12208

[8] Parmesan C. Ecological and Evolutionary Responses to Recent Climate Change. Annu Rev Ecol Evol Syst. 2006; 37: 637–669. 10.1146/annurev.ecolsys.37.091305.110100

[9] Stange EE, Ayres MP. Climate Change Impacts: Insects In: Encyclopedia of Life Sciences (ELS). Chichester: John Wiley & Sons, Ltd; 2010 10.1002/9780470015902.a0022555

[10] Bellard C, Bertelsmeier C, Leadley P, Thuiller W, Courchamp F. Impacts of climate change on the future of biodiversity. Ecol Lett. 2012; 15: 365–377. 10.1111/j.1461-0248.2011.01736.x 22257223 PMC3880584

[11] Parmesan C, Yohe G. A globally coherent fingerprint of climate change impacts across natural systems. Nature. 2003; 421: 37–42. 10.1038/nature01286 12511946

[12] Massot M, Clobert J, Ferrière R. Climate warming, dispersal inhibition and extinction risk. Glob Chang Biol. 2008; 14: 461–469. 10.1111/j.1365-2486.2007.01514.x

[13] Walther GR, Post E, Convey P, Menzel A, Parmesan C, Beebee TJC, et al Ecological responses to recent climate change. Nature. 2002; 416: 389–395. 10.1038/416389a 11919621

[14] Dobrowski SZ, Abatzoglou J, Swanson AK, Greenberg JA, Mynsberge AR, Holden ZA, et al The climate velocity of the contiguous United States during the 20th century. Glob Chang Biol. 2013; 19: 241–251. 10.1111/gcb.12026 23504735

[15] Anderegg WRL, Hicke JA, Fisher RA, Allen CD, Aukema J, Bentz B, et al Tree mortality from drought, insects, and their interactions in a changing climate. New Phytol. 2015; 208: 674–683. 10.1111/nph.13477 26058406

[16] Pereira HM, Leadley PW, Proença V, Alkemade R, Scharlemann PW, Fernandez- Manjarrés JF, et al Scenarios for Global Biodiversity in the 21st Century. Science. 2010; 330: 1496–1501. 10.1126/science.1196624 20978282

[17] Pimm SL, Jenkins CN, Abell R, Brooks TM, Gittleman JL, Joppa LN. The biodiversity of species and their rates of extinction, distribution, and protection. Science. 2014; 344 (6187): 1246752 10.1126/science.1246752 24876501

[18] Meyers LA, Bull JJ. Fighting change with change. Trends Ecol Evol. 2002; 17 (12): 551–557. 10.1016/S0169-5347(02)02633-2

[19] Botkin DB, Saxe H, Araújo MB, Betts R, Bradshaw RHW, Cedhagen T, et al Forecasting the Effects of Global Warming on Biodiversity. BioScience. 2007; 57 (3): 227–236. 10.1641/B570306

[20] Thuiller W, Araújo MB, Lavorel S. Do we need land-cover data to model species distributions in Europe? J Biogeogr. 2004; 31: 353–361. 10.1046/j.0305-0270.2003.00991.x

[21] Boggs CL. The fingerprints of global climate change on insect populations. Curr Opin Insect Sci. 2016; 17: 69–73. 10.1016/j.cois.2016.07.004 27720076

[22] Bubová T, Vrabec V, Kulma M, Nowicki P. Land management impacts on European butterflies of conservation concern: a review. J Insect Conserv. 2015; 19: 805–821. 10.1007/s10841-015-9819-9

[23] Fourcade Y, Ranius T, Öckinger E. Temperature drives abundance fluctuations, but spatial dynamics is constrained by landscape configuration: Implications for climate-driven range shift in a butterfly. J Anim Ecol. 2017; 86: 1339–1351. 10.1111/1365-2656.12740 28796909

[24] Newbold T, Hudson NL, Hill S, Contu S, Lysenko I, Senior RA, et al Global effects of land use on local terrestrial biodiversity. Nature. 2015; 520: 45–50. 10.1038/nature14324 25832402

[25] Lomolino MV. Conservation biogeography In: Lomolino MV, Heaney LR, editors. Frontiers of Biogeography: New Directions in the Geography of Nature. Sunderland, Massachusetts: Sinauer Associates; 2004 pp. 293–296.

[26] Cardoso P, Erwin TL, Borges PA, New TR. The seven impediments in invertebrate conservation and how to overcome them. Biol Conserv. 2011; 144: 2647–2655. 10.1016/j.biocon.2011.07.024

[27] Elith J, Graham CH, Anderson RP, Dudik M, Ferrier S, Guisan A, et al Novel methods improve prediction of species’ distributions from occurrence data. Ecography. 2006; 29: 129–151. 10.1111/j.2006.0906-7590.04596.x

[28] Peterson AT. Uses and requirements of ecological niche models and related distributional models. Biodivers Inform. 2006; 3: 59–72. 10.17161/bi.v3i0.29

[29] Thuiller W, Albert C, Araújo M, Berry P, Cabeza M, Guisan A, et al Predicting global change impacts on plant species’ distributions: future challenges. Perspect Plant Ecol Evol Syst. 2008; 9: 137–152. 10.1016/j.ppees.2007.09.004

[30] Ramsfield TD, Bentz BJ, Faccoli M, Jactel H, Brockerhoff EG. Forest health in a changing world: effects of globalization and climate change on forest insect and pathogen impacts. Forestry. 2016; 89: 245–252. 10.1093/forestry/cpw018

[31] Wang R, Li Q, He S, Liu Y, Wang M, Jiang G. Modeling and mapping the current and future distribution of *Pseudomonas syringae* pv. *actinidiae* under climate change in China. PloS ONE. 2018; 13: e019215 10.1371/journal.pone.0192153 29389964 PMC5794145

[32] Romo H, Silvestre M, Munguira ML. Potential distribution models and the effect of climatic change on the distribution of *Phengaris nausithous* considering its food plant and host ants. J Insect Conserv. 2015; 19: 1101–1118. 10.1007/s10841-015-9825-y

[33] Barrows CW, Rotenberry JT, Allen MF. Assessing sensitivity to climate change and drought variability of a sand dune endemic lizard. Biol Conserv. 2010; 143: 731–736. 10.1016/j.biocon.2009.12.013

[34] Kaloveloni A, Tscheulin T, Vujić A, Radenković S, Petanidou T. Winners and losers of climate change for the genus *Merodon* (Diptera: Syrphidae) across the Balkan Peninsula. Ecol Model. 2015; 313: 201–211. 10.1016/j.ecolmodel.2015.06.032

[35] Miličić M, Vujić A, Jurca T, Cardoso P. Designating conservation priorities for Southeast European hoverflies (Diptera: Syrphidae) based on species distribution models and species vulnerability. Insect Conserv Divers. 2017; 10 (4): 354–366. 10.1111/icad.12232

[36] Potts SG, Biesmeijer JC, Kremen C, Neumann P, Schweiger O, Kunin WE. Global pollinator declines: trends, impacts and drivers. Trends Ecol Evol. 2010; 25 (6): 345–353. 10.1016/j.tree.2010.01.007 20188434

[37] Radenković S, Schweiger O, Milić D, Harpke A, Vujić A. Living on the edge: Forecasting the trends in abundance and distribution of the largest hoverfly genus (Diptera: Syrphidae) on the Balkan Peninsula under future climate change. Biol Conserv. 2017; 212: 216–229. 10.1016/j.biocon.2017.06.026

[38] Schweiger O, Musche M, Bailey D, Billeter R, Diekotter T, Hendrickx F, et al Functional richness of local hoverfly communities (Diptera, Syrphidae) in response to land use across temperate Europe. Oikos. 2007; 116: 461–472. 10.1111/j.2007.0030-1299.15372.x PMC7194119 32367896

[39] Becher MA, Osborne JL, Thorbek P, Kennedy PJ, Grimm V. Towards a systems approach for understanding honeybee decline: a stocktaking and synthesis of existing models. J Appl Ecol. 2013; 50: 868–880. 10.1111/1365-2664.12112 . 24223431 PMC3810709

[40] Kerr JT, Pindar A, Galpern P, Packer L, Potts SG, Roberts SM, et al Climate change impacts on bumblebees converge across continents. Science. 2015; 349: 177–180. 10.1126/science.aaa7031 . 26160945

[41] Aguirre-Gutiérrez J, Kissling WD, Biesmeijer JC, WallisDeVries MF, Reemer M, Carvalheiro LG. Historical changes in the importance of climate and land use as determinants of Dutch pollinator distributions. J Biogeogr. 2017; 44: 696–707. 10.1111/jbi.12937

[42] Araújo M, Alagador D, Cabeza M, Nogués-Bravo D, Thuiller W. Climate change threatens European conservation areas. Ecol Lett. 2011; 14: 484–492. 10.1111/j.1461-0248.2011.01610.x 21447141 PMC3116148

[43] Carvalho SB, Brito JC, Crespo EJ, Possingham H. From climate change predictions to actions–conserving vulnerable animal groups in hotspots at a regional scale. Glob Chang Biol. 2010; 16: 3257–3270. 10.1111/j.1365-2486.2010.02212.x

[44] Silva DP, Spigoloni ZA, Camargos LM, de Andrade AFA, De Marco P Jr, Engel MS. Distributional modeling of Mantophasmatodea (Insecta: Notoptera): a preliminary application and the need for future sampling. Org Divers Evol. 2016; 16: 259–268. 10.1007/s13127-015-0250-6

[45] Silva DP, Aguiar AG, Simião-Ferreira J. Assessing the distribution and conservation status of a long-horned beetle with species distribution models. J Insect Conserv. 2016; 20: 611–620. 10.1007/s10841-016-9892-8

[46] Vujić A, Radenković S, Nikolić T, Radišić D, Trifunov S, Andrić A, et al Prime Hoverfly (Insecta: Diptera: Syrphidae) Areas (PHA) as conservation tool in Serbia. Biol Conserv 2016; 198: 22–32. 10.1016/j.biocon.2016.03.032

[47] Carvalheiro GL, Kunin WE, Keil P, Aguirre-Gutiérrez J, Ellis WN, Fox R, et al Species richness declines and biotic homogenisation have slowed down for NW-European pollinators and plants. Ecol Lett. 2013; 16: 870–878. 10.1111/ele.12121 23692632 PMC3738924

[48] Miličić M, Vujić A, Cardoso P. Effects of climate change on the distribution of hoverfly species (Diptera: Syrphidae) in Southeast Europe. Biodivers Conserv. 2018; 27 (5): 1173–1187. 10.1007/s10531-017-1486-6

[49] Vujić A, Ståhls G, Ačanski J, Bartsch H, Bygebjerg R., Stefanović A. Systematics of Pipizini and taxonomy of European *Pipiza* Fallén: molecular and morphological evidence (Diptera, Syrphidae). Zool Scr. 2013; 42 (3): 288–305. 10.1111/zsc.12005

[50] Nikolić T, Radišić D, Milić D, Marković V, Trifunov S, Jovičić S, et al Models of the potential distribution and habitat preferences of the genus *Pipiza* (Syrphidae: Diptera) on the Balkan Peninsula. Arch Biol Sci. 2013; 65 (3): 1037–1052. 10.2298/ABS1303037N

[51] Speight MCD. Species accounts of European Syrphidae, 2018 In: Speight MCD, Castella E, Sarthou JP, Vanappelghem C, editors. Syrph the Net: the database of European Syrphidae (Diptera). Vol. 103 Dublin: Syrph the Net publications; 2018.

[52] Cardoso P. Red: IUCN Redlisting Tools. R Package Version 0.1.0. CRAN, Vienna, Austria; 2016.

[53] R Development Core Team. R: A Language and Environment for Statistical Computing. R Foundation for Statistical Computing, Vienna, Austria; 2016. https://www.R-project.org/

[54] Hijmans RJ, Cameron SE, Parra JL, Jones PG, Jarvis A. Very high resolution interpolated climate surfaces for global land areas. Int J Climatol. 2005; 25: 1965–1978. 10.1002/joc.1276

[55] Van Vuuren DP, Edmonds J, Kainuma M, Riahi K, Thomson A, Hibbard K, et al The representative concentration pathways: an overview. Clim Change. 2011; 109: 5–31. 10.1007/s10584-011-0148-z

[56] IPCC. Fifth assessment report (AR5). Cambridge: Cambridge University Press; 2014.

[57] Van Vuuren D, den Elzen M, Lucas P, Eickhout B, Strengers B, van Ruijven B, et al Stabilizing greenhouse gas concentrations at low levels: an assessment of reduction strategies and costs. Clim Change. 2007; 81: 119–159. 10.1007/s10584-006-9172-9

[58] Clarke L, Edmonds J, Jacoby H, Pitcher H, Reilly J, Richels R. Scenarios of Greenhouse Gas Emissions and Atmospheric Concentrations. Sub-report 2.1A of Synthesis and Assessment Product 2.1 by the U.S. Climate Change Science Program and the Subcommittee on Global Change Research. Washington DC, USA: Department of Energy, Office of Biological & Environmental Research; 2007.

[59] Fujino J, Nair R, Kainuma M, Masui T, Matsuoka Y. Multi-gas mitigation analysis on stabilization scenarios using AIM global model. Energy J. Special Issue (Multi-Greenhouse Gas Mitigation and Climate Policy) 2006; 27(3): 343–354.

[60] Tongwe WU, Lianchun S, Weipin LI, Zaizh W, Hu Z, Xiaog X, et al An Overview of BCC Climate System Model Development and Application for Climate Change Studies. J Meteorol Res. 2014; 28: 34–56. 10.1007/s13351-014-3041-7

[61] Gent PR, Danabasoglu G, Donner LJ, Holland MM, Hunke EC, Jayne SR, et al The Community Climate System Model Version 4. J Clim. 2011; 24: 4973–4991. 10.1175/2011JCLI4083.1

[62] Jones CD, Hughes JK, Bellouin N, Hardiman SC, Jones GS, Knight JS. et al The HadGEM2-ES implementation of CMIP5 centennial simulations. Geosci Model Dev. 2011; 4: 543–570. 10.5194/gmd-4-543-2011

[63] EEA European Environment Agency. Digital elevation model over Europe (EU-DEM); 2013. [accessed 2015 Nov 29] http://www.eea.europa.eu/data-and-maps/data/eu-dem.

[64] Hijmans RJ, Guarino L, Mathur P. DIVA-GIS, version 7.5. A geographic information system for the analysis of species distribution data. Manual. 2012.

[65] Dell Inc. Dell Statistica (data analysis software system), version 13. 2016. http://www.software.dell.com

[66] Corine Land Cover, CLC. 2012. [accessed 2018 Aug 2] http://land.copernicus.eu/pan-european/corine-land-cover/clc-2012.

[67] Naimi B usdm: Uncertainty Analysis for Species. Distribution models. R package version 1. 2015; 1–15.

[68] Phillips SJ, Anderson RP, Schapire RE. Maximum Entropy Modeling of Species Geographic Distributions. Ecol Model. 2006; 190: 231–259. 10.1016/j.ecolmodel.2005.03.026

[69] Phillips SJ, Dudik M. Modeling of species distributions with Maxent: new extensions and a comprehensive evaluation. Ecography. 2008; 31: 161–175. 10.1111/j.0906-7590.2008.5203.x

[70] Merow C, Smith MJ, Silander JA Jr. A practical guide to MaxEnt for modeling species’ distributions: what it does, and why inputs and settings matter. Ecography. 2013; 36: 1058–1069. 10.1111/j.1600-0587.2013.07872.x

[71] Fourcade Y, Engler JO, Rödder D, Secondi J. Mapping Species Distributions with MAXENT Using a Geographically Biased Sample of Presence Data: A Performance Assessment of Methods for Correcting Sampling Bias. PLoS ONE. 2014; 9 (5): e97122 10.1371/journal.pone.0097122 24818607 PMC4018261

[72] Elith J, Phillips SJ, Hastie T, Dudík M, Chee YE, Yates CJ. A statistical explanation of MaxEnt for ecologists. Divers Distrib. 2011; 17: 43–57. 10.1111/j.1472-4642.2010.00725.x

[73] Guisan A, Tingley R, Baumgartner JB, Naujo-Kaitis‐Lewis I, Sutcliffe PR, Tulloch AI, et al Predicting species distributions for conservation decisions. Ecol Lett. 2013; 16: 1424–1435. 10.1111/ele.12189 24134332 PMC4280402

[74] Urbani F, D’Alessandro P, Frasca R, Biondi M. Maximum entropy modeling of geographic distributions of the flea beetle species endemic in Italy (Coleoptera: Chrysomelidae: Galerucinae: Alticini). Zool Anz. 2015; 258: 99–109. 10.1016/j.jcz.2015.08.002

[75] Breiman L, Friedman J, Stone CJ, Olshen RA. Classification and Regression Trees. London, UK: Chapman & Hall; 1984.

[76] Nix HA. A biogeographic analysis of Australian elapid snakes In: Longmore R, editor. Atlas of elapid snakes of Australia. Australian Flora and Fauna Series 7. Canberra, Australia: Australian Government Publishing Service; 1986 pp. 4–15.

[77] McCullagh P, Nelder JA. Generalized Linear Models. London, UK: Chapman & Hall; 1989.

[78] Ripley BD. Pattern Recognition and Neural Networks. Cambridge, UK: Cambridge University Press; 1996.

[79] Verbruggen H, Tyberghein L, Belton GS, Mineur F, Jueterbock A, Hoarau G, et al Improving Transferability of Introduced Species’ Distribution Models: New Tools to Forecast the Spread of a Highly Invasive Seaweed. PLoS ONE. 2013; 8 (6): e68337 10.1371/journal.pone.0068337 23950789 PMC3732097

[80] Muscarella R, Galante PJ, Soley-Guardia M, Boria RA, Kass JM, Uriarte M, et al ENMeval: An R package for conducting spatially independent evaluations and estimating optimal model complexity for Maxent ecological niche models. Methods Ecol Evol. 2014; 5: 1198–1205. 10.1111/2041-210X.12261

[81] Burnham KP, Anderson DR. Multimodel Inference: Understanding AIC and BIC in Model Selection. Sociol Methods Res. 2004; 33: 261–304. 10.1177/0049124104268644

[82] Warren DL, Seifert SN. Ecological niche modeling in Maxent: the importance of model complexity and the performance of model selection criteria. Ecol Appl. 2011; 21: 335–342. 10.2307/29779663 21563566

[83] Liu C, Berry PM, Dawson TP, Pearson RG. Selecting thresholds of occurrence in the prediction of species distributions. Ecography. 2005; 28: 385–393. 10.1111/j.0906-7590.2005.03957.x

[84] Liu C, White M, Newell G. Selecting thresholds for the prediction of species occurrence with presence-only data. J Biogeogr. 2013; 40 (4): 778–789. 10.1111/jbi.12058

[85] Peterson AT, Ortega-Huerta MA, Bartley J, Sánchez-Cordero V, Soberón J, Buddemeier RH, et al Future projections for Mexican faunas under global climate change scenarios. Nature. 2002; 416: 626–629. 10.1038/416626a 11948349

[86] Thuiller W. Patterns and uncertainties of species’ range shifts under climate change. Glob Chang Biol. 2004; 10 (12): 2020–2027. 10.1111/j.1365-2486.2004.00859.x PMC4340562 25200514

[87] Broennimann O, Thuiller W, Hughes G, Midgley GF, Alkemade JMR, Guisan A. Do geographic distribution, niche property and life form explain plants’ vulnerability to global change? Glob Chang Biol. 2006; 12(6): 1079–1093. 10.1111/j.1365-2486.2006.01157.x

[88] Wisz MS, Hijmans RJ, Li J, Peterson AT, Graham CH, Guisan A, NCEAS Predicting Species Distributions Working Group. Effects of sample size on the performance of species distribution models. Diversity and Distributions. 2008, 14(5): 763–773. 10.1111/j.1472-4642.2008.00482.x

[89] Andrews T, Gregory JM, Webb MJ, Taylor KE. Forcing, feedbacks and climate sensitivity in CMIP5 coupled atmosphere-ocean climate models. Geophys Res Lett. 2012; 39 (9): L09712 10.1029/2012GL051607

[90] Bitz CM, Shell KM, Gent PR, Bailey DA, Danabasoglu G, Armour KC, et al Climate sensitivity of the Community Climate System Model, version 4. J Clim. 2012; 25: 3053–3070. 10.1175/JCLI-D-11-00290.1

[91] Meehl GA, Washington WM, Arblaster JM, Hu A, Teng H, Tebaldi C, et al Climate system response to external forcings and climate change projections in CCSM4. J Clim. 2012; 25: 3661–3683. 10.1175/JCLI-D-11-00240.1

[92] Meraner K, Mauritsen T, Voigt A. Robust increase in equilibrium climate sensitivity under global warming. Geophys Res Lett. 2013; 40: 5944–5948. 10.1002/2013GL058118

[93] Dixon AFG, Honěk A, Keil P, Ali M, Kotela A, Šizling AL, et al Relationship between the minimum and maximum temperature thresholds for development in insects. Functional Ecology. 2009; 23 (2): 257–264. 10.1111/j.1365-2435.2008.01489.x

[94] Allen CD, Macalady AK, Chenchouni H, Bachelet D, McDowell N, Vennetier M, et al A global overview of drought and heat-induced tree mortality reveals emerging climate change risks for forests. For Ecol Manage. 2010; 259 (4): 660–684. 10.1016/j.foreco.2009.09.001

[95] Breda N, Huc R, Granier A, Dreyer E. Temperate forest trees and stands under severe drought: a review of ecophysiological responses, adaptation processes and long-term consequences. Ann For Sci. 2006; 63: 625–644. 10.1051/forest:2006042

[96] Landmann G, Dreyer E, editors. Impacts of drought and heat on forest. Synthesis of available knowledge, with emphasis on the 2003 event in Europe. Ann For Sci (Special Issue). 2006; 3 (6): 567–652.

[97] Parmesan C, Ryrholm N, Stefanescu C, Hill JK, Thomas CD, Descimon H, et al Poleward shifts in geographical ranges of butterfly species associated with regional warming. Nature. 1999; 399: 579–583. 10.1038/21181

[98] Hickling R, Roy DB, Hill JK, Thomas CD. A northward shift of range margins in British Odonata. Glob Chang Biol. 2005; 11 (3): 502–506. 10.1111/j.1365-2486.2005.00904.x

[99] Settele J, Kudrna O, Harpke A, Kühn I, van Swaay C, Verovnik R, et al Climatic Risk Atlas of European Butterflies. Biorisk 1 (Special Issue), Bulgaria: Pensoft Publishers; 2008.

[100] Heikkinen RK, Luoto M, Leikola N, Pöyry J, Settele J, Kudrna O, et al Assessing the vulnerability of European butterflies to climate change using multiple criteria. Biodivers Conserv. 2010; 19: 695–723. 10.1007/s10531-009-9728-x

[101] Vanhanen H, Veteli TO, Päivinen S, Kellomäki S, Niemelä P. Climate change and range shifts in two insect defoliators: gypsy moth and nun moth–a model study. Silva Fennica. 2007; 41 (4): 621–638.

[102] Roth T, Plattner M, Amrhein V. Plants, Birds and Butterflies: Short-Term Responses of Species Communities to Climate Warming Vary by Taxon and with Altitude. PLoS ONE. 2014; 9 (1): e82490 10.1371/journal.pone.0082490 24416144 PMC3885385

[103] Obregón R, Fernández Haeger J, Jordano D. Effects of climate change on three species of Cupido (Lepidoptera, Lycaenidae) with different biogeographic distribution in Andalusia, southern Spain. Anim Biodivers Conserv. 2016; 39 1: 115–128.

[104] Warren MS, Hill JK, Thomas JA, Asher J, Fox R, Huntley B, et al Rapid responses of British butterflies to opposing forces of climate and habitat change. Nature. 2001; 414: 65–69. 10.1038/35102054 11689943

[105] Hof AR, Svahlin A. The potential effect of climate change on the geographical distribution of insect pest species in the Swedish boreal forest. Scandinavian Journal of Forest Research. 2016; 31 (1): 29–39. 10.1080/02827581.2015.1052751

[106] Rasmont P, Franzén M, Lecocq T, Harpke A, Roberts SPM, Biesmeijer JC, et al Climatic Risk and Distribution Atlas of European Bumblebees. Biorisk 10 (Special Issue), Bulgaria: Pensoft Publishers; 2015 10.3897/biorisk.10.4749

[107] Harrington R, Clark SJ, Welham SJ, Verrier PJ, Denholm CH, Hullé M, et al Environmental change and the phenology of European aphids. Glob Chang Biol. 2007; 13: 1550–1564. 10.1111/j.1365-2486.2007.01394.x

[108] Régnière J. Predicting insect continental distributions from species physiology. Unasylva. 2009; 60 (231): 37–42.

[109] Harrison PA, Berry PM, Butt N, New M. Modelling climate change impacts on species’ distributions at the European scale: implications for conservation policy. Environ Sci Policy. 2006; 9: 116–128. 10.1016/j.envsci.2005.11.003

[110] Wilson RJ, Maclean IMD. Recent evidence for the climate change threat to Lepidoptera and other insects. J Insect Conserv. 2011; 15: 259–268. 10.1007/s10841-010-9342-y

[111] van Baaren J, Le Lann C, van Alphen JJM. Consequences of Climate Change for Aphid-Based Multi-trophic Systems In: Kindlmann P, et al, editors. Aphid Biodiversity under Environmental Change. Springer Science+Business Media B.V.; 2010 pp. 55–68. 10.1007/978-90-481-8601-3_4

[112] Araújo MB, Rahbek C. How does climate change affect biodiversity? Science. 2006; 313: 1396–1397. 10.1126/science.1131758 16959994

[113] Ballesteros-Mejia L, Kitching IJ, Jetz W, Beck J. Putting insects on the map: near-global variation in sphingid moth richness along spatial and environmental gradients. Ecography. 2017; 40: 698–708. 10.1111/ecog.02438

