# Supplementary figures and images for: Stability and changes in the distribution of Pipiza hoverflies (Diptera, Syrphidae) in Europe under projected future climate conditions
Source: PLoS One. 2019 Sep 4;14(9):e0221934. doi: 10.1371/journal.pone.0221934 (PMC6726199; doi:10.1371/journal.pone.0221934)

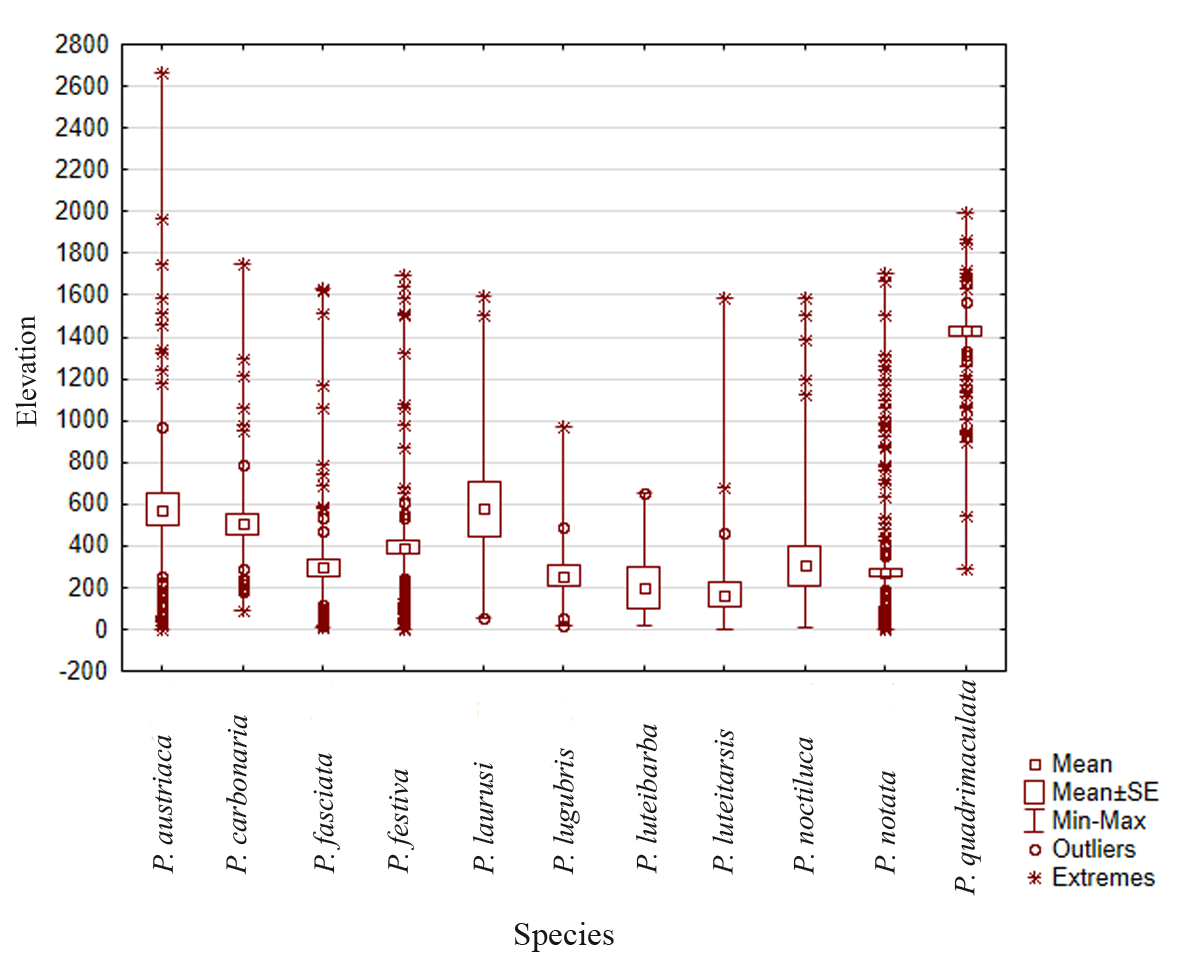

Supplement: S1 Fig — (TIF) [file pone.0221934.s001.tif]
