# Supplementary material for: Stability and changes in the distribution of Pipiza hoverflies (Diptera, Syrphidae) in Europe under projected future climate conditions
Source: PLoS One. 2019 Sep 4;14(9):e0221934. doi: 10.1371/journal.pone.0221934 (PMC6726199; doi:10.1371/journal.pone.0221934)
Supplement: S1 Appendix — (DOCX) [file pone.0221934.s002.docx]

**S1 Appendix**. **List of collections were the material used in this study was deposited**

AEU - University of the Aegean, Mytiline, (Greece); BMNH – Natural History Museum, London, UK; CEUA - Colección Entomológica de la Universidad de Alicante,Alicante, Spain; FSUNS – Faculty of Sciences, Department of Biology and Ecology, University of Novi Sad, Serbia; GLAHM - Hunterian Zoology Museum, University of Glasgow, Glasgow, Scotland, UK; HMLD - Darmstadt museum, Darmstadt, Germany;HPM – Croatian Natural History Museum, Zagreb; MACMUSNH - Macedonian Museum of Natural History, Skoplje, (FRY of Macedonia); MNCN - Museo Nacional de Ciencias Naturales, Madrid, (Spain); MNHN – Musee National d’Histoire Naturelle, Paris, France; MNHP - National Museum Prague, Czech republic; MZH – Zoological Museum of The Finnish Museum of Natural History, Helsinki, Finland; NMNHS - National Museum of Natural History, Sofia, (Bulgaria); NHMBEO – Prirodnjački muzej Beograd, Serbia; NHMW – Naturhistorisches Museum Wien, Austria; RMNH – Natural Museum of Natural History, Leiden, Netherlands; SAR – Zemaljski Muzej Sarajevo, Bosnia-Herzegovina; SMNS - Stuttgart State Museum of Natural History, Stuttgart, (Germany); TAU – Tel Aviv University, Israel; UASK – Ukrainian Academy of Sciences, Schmalhausen Institute of Zoology, Kiev, Ukraine; WML – World Museum Liverpool, UK; ZHMB – Zoologisches Museum of the Humboldt University, Berlin, Germany; ZMC – Zoological Museum, Natural History Museum of Denmark, University of Copenhagen, Copenhagen, Denmark; ZFMK - Zoological Research Museum Alexander Koenig, Bon, Germany; WML - World Museum Liverpool, UK.
