# Supplementary material for: Stability and changes in the distribution of Pipiza hoverflies (Diptera, Syrphidae) in Europe under projected future climate conditions
Source: PLoS One. 2019 Sep 4;14(9):e0221934. doi: 10.1371/journal.pone.0221934 (PMC6726199; doi:10.1371/journal.pone.0221934)
Supplement: S1 Table — (DOCX) [file pone.0221934.s003.docx]

S1 Table. Numbers of records for analysed species and respective variables used to develop the model and their percentage contribution

|  | | Pa | Pc | Pfa | Pfe | Plg | Plu | Pnc | Pn | Pq |
| --- | --- | --- | --- | --- | --- | --- | --- | --- | --- | --- |
| Environmental variables | No. of records | 40 | 16 | 37 | 53 | 63 | 26 | 111 | 61 | 86 |
| Elevation | | X |  |  |  |  |  | X |  | X |
| Annual Mean Temperature (BIO1) | |  |  | X | X |  |  |  |  |  |
| **Mean Diurnal Range (**BIO2) | | X | X |  | X | X | X | X | X | X |
| Isothermality (BIO3) | |  |  |  |  |  |  |  |  |  |
| Temperature Seasonality (BIO4) | | X | X |  |  |  |  | X |  |  |
| **Max temperature of warmest month (BIO5)** | |  |  |  |  |  |  |  |  |  |
| **Min Temperature of Coldest Month (**BIO6) | |  |  |  |  |  |  |  |  |  |
| Temperature annual range (BIO7) | |  |  |  |  |  |  |  |  |  |
| Mean Temperature of Wettest Quarter (BIO8) | | X |  | X |  | X | X | X | X | X |
| Mean Temperature of Driest Quarter (BIO9) | | X |  |  | X |  |  |  |  | X |
| Mean Temperature of Warmest Quarter (BIO10) | |  |  |  |  |  |  |  |  |  |
| Mean temperature of coldest quarter (BIO11) | |  |  |  |  |  |  |  |  |  |
| Annual precipitation (BIO12) | |  |  |  |  |  |  |  |  |  |
| Precipitation of wettest month (BIO13) | |  |  |  | X |  |  |  |  | X |
| Precipitation of driest month (BIO14) | |  |  |  |  |  |  |  |  |  |
| **Precipitation Seasonality** (BIO15) | | X | X | X | X | X | X | X | X | X |
| Precipitation of wettest quarter (BIO16) | |  |  |  |  |  |  |  |  |  |
| Precipitation of driest quarter (BIO17) | |  |  |  |  |  |  |  |  |  |
| **Precipitation of Warmest Quarter (**BIO18) | |  |  | X |  | X |  | X | X |  |
| Precipitation of Coldest Quarter (BIO19) | |  |  |  |  |  |  |  |  |  |
| Distance from forest | | X | X |  | X |  | X | X | X |  |
| Proportion of the grid cell covered with grassland | | X |  | X |  | X | X | X | X |  |
| Proportion of the grid cell covered with forest | | X | X | X | X | X | X | X | X | X |
| Proportion of the grid cell covered with agricultural land | |  |  | X |  | X |  | X | X | X |

Pa - *P. austriaca, Pc -* P. *carbonaria, Pfa* - *P. fasciata,* Pfe - *P. festiva*, Plg - *P. lugubri,* Plu - *P. luteitarsis,* Pnc - *P. noctiluca*, Pn - *P. notata,* Pq - *P. quadrimaculata*
