# Supplementary material for: Stability and changes in the distribution of Pipiza hoverflies (Diptera, Syrphidae) in Europe under projected future climate conditions
Source: PLoS One. 2019 Sep 4;14(9):e0221934. doi: 10.1371/journal.pone.0221934 (PMC6726199; doi:10.1371/journal.pone.0221934)
Supplement: S2 Table — (DOCX) [file pone.0221934.s004.docx]

S2 Table. Model comparison based on Akaike Information Criteria (AICc)

| **Species** | **Feature types** | **Regularization Multiplier** | **AUC** | **Parameters** | **AUCtest** | **Kappa** | **Treshold** |
| --- | --- | --- | --- | --- | --- | --- | --- |
| *P. austriaca* | L | 2 | 0.716 | 8 | 0.746 | 0.7666934 | 0.498 |
| *P. carbonaria* | H | 3 | 0.789 | 3 | 0.830 | 0.6848673 | 0.526 |
| *P. fasciata* | H | 3 | 0.721 | 8 | 0.703 | 0.7469238 | 0.539 |
| *P. festiva* | LQ | 1 | 0.703 | 9 | 0.723 | 0.8202943 | 0.476 |
| *P. lugubris* | H | 2.5 | 0.811 | 15 | 0.759 | 0.8582099 | 0.437 |
| *P. luteitarsis* | L | 3.5 | 0.820 | 4 | 0.832 | 0.7556499 | 0.498 |
| *P. noctiluca* | H | 4 | 0.744 | 15 | 0.736 | 0.8642240 | 0.477 |
| *P. notata* | H | 3 | 0.808 | 17 | 0.759 | 0.9667847 | 0.527 |
| *P. quadrimaculata* | LQHP | 3.5 | 0.816 | 25 | 0.754 | 0.9790636 | 0.445 |

Feature classes: L - linear, P - product, Q - quadratic, H - hinge
