# Supplementary material for: Stability and changes in the distribution of Pipiza hoverflies (Diptera, Syrphidae) in Europe under projected future climate conditions
Source: PLoS One. 2019 Sep 4;14(9):e0221934. doi: 10.1371/journal.pone.0221934 (PMC6726199; doi:10.1371/journal.pone.0221934)
Supplement: S3 Table — (DOCX) [file pone.0221934.s005.docx]

S3 Table. Percentages of range reduction/expansion relative to current distributions for species of the genus *Pipiza* in Europe based on the BCC_CSM1.1, CCSM4 and HadGEM2-ES climate model predictions and for different climate change scenarios.

|  | 2050 | | | | 2070 | | | | Tendency |
| --- | --- | --- | --- | --- | --- | --- | --- | --- | --- |
|  | 2.6 | 4.5 | 6.0 | 8.5 | 2.6 | 4.5 | 6.0 | 8.5 |  |
| BCC_CSM1.1 |  |  |  |  |  |  |  |  |  |
| *P. austriaca* | -6.37 | -37.52 | 4.39 | -12.78 | 3.01 | 8.87 | -7.35 | -14.07 | R |
| *P. carbonaria* | 15.35 | 9.17 | 18.56 | 14.65 | 14.75 | 27.83 | 17.42 | 16.19 | E |
| *P. fasciata* | -20.48 | -25.89 | -28.87 | -22.31 | -27.47 | -31.13 | -26.43 | -25.02 | R |
| *P. festiva* | -50.06 | -47.07 | -49.90 | -36.36 | -46.10 | -46.78 | -30.94 | -24.17 | R |
| *P. lugubris* | -7.56 | -9.69 | -12.60 | -19.02 | -6.52 | -5.87 | -19.57 | -20.99 | R |
| *P. luteitarsis* | -17.37 | -19.71 | -22.00 | -17.27 | -24.74 | -25.45 | -21.07 | -17.60 | R |
| *P. noctiluca* | 2.96 | 4.88 | 11.70 | 4.42 | 9.93 | 19.09 | 5.96 | 3.08 | E |
| *P. notata* | 5.01 | 1.14 | 2.31 | 2.32 | 3.44 | 6.68 | 2.89 | 7.06 | E |
| *P. quadrimaculata* | 11.16 | 3.19 | 0.19 | -5.69 | 8.17 | 12.03 | -5.31 | -7.74 | E-R |
| CCSM4 |  |  |  |  |  |  |  |  |  |
| *P. austriaca* | -10.53 | -8.28 | -10.29 | -11.93 | -14.38 | -8.38 | -13.15 | -22.53 | R |
| *P. carbonaria* | -5.31 | -8.19 | -11.12 | -10.11 | -5.93 | -6.81 | -6.81 | -19.99 | R |
| *P. fasciata* | -53.84 | -49.62 | -54.56 | -44.97 | -17.20 | -18.21 | -17.57 | -18.67 | R |
| *P. festiva* | -53.84 | -49.62 | -54.56 | -44.97 | -54.21 | -50.82 | -48.75 | -39.40 | R |
| *P. lugubris* | -8.77 | -19.26 | -20.05 | -26.71 | -7.79 | -20.07 | -21.70 | -39.30 | R |
| *P. luteitarsis* | -40.88 | -16.21 | -16.34 | -14.89 | -13.12 | -14.29 | -13.48 | -13.82 | R |
| *P. noctiluca* | -13.77 | -15.13 | -16.18 | -18.45 | -13.38 | -16.41 | -18.05 | -27.59 | R |
| *P. notata* | -0.49 | -7.85 | -11.63 | -16.91 | 3.61 | -12.53 | -9.73 | -23.55 | R |
| *P. quadrimaculata* | -3.18 | -13.49 | -15.01 | -16.50 | -4.92 | -16.55 | -13.33 | -25.69 | R |
| HadGEM2-ES |  |  |  |  |  |  |  |  |  |
| *P. austriaca* | -11.47 | -17.95 | -10.77 | -25.72 | -4.30 | -18.11 | -19.11 | -47.41 | R |
| *P. carbonaria* | -4.42 | -3.21 | -3.22 | -9.37 | 1.42 | -7.26 | -6.98 | -30.37 | R |
| *P. fasciata* | -18.77 | -20.22 | -17.33 | -17.88 | -19.18 | -17.95 | -19.52 | -29.31 | R |
| *P. festiva* | -40.56 | -24.79 | -36.94 | -19.12 | -37.43 | -20.47 | -23.22 | -3.50 | R |
| *P. lugubris* | -24.57 | -34.90 | -28.99 | -39.06 | -24.21 | -42.13 | -38.07 | -59.55 | R |
| *P. luteitarsis* | -15.16 | -16.56 | -12.34 | -13.96 | -14.35 | -15.56 | -16.31 | -24.85 | R |
| *P. noctiluca* | -13.04 | -17.12 | -14.05 | -22.61 | -7.59 | -18.18 | -18.65 | -42.76 | R |
| *P. notata* | -11.09 | -19.14 | -16.45 | -20.48 | -13.25 | -27.69 | -24.24 | -43.03 | R |
| *P. quadrimaculata* | -15.59 | -23.77 | -22.88 | -22.87 | -24.35 | -13.56 | -27.96 | -24.38 | R |

R - reduction, E - expansion
